# An Easily Fabricated High Performance Fabry-Perot Optical Fiber Humidity Sensor Filled with Graphene Quantum Dots

**DOI:** 10.3390/s21030806

**Published:** 2021-01-26

**Authors:** Ning Wang, Wenhao Tian, Haosheng Zhang, Xiaodan Yu, Xiaolei Yin, Yonggang Du, Dailin Li

**Affiliations:** College of Science, China University of Petroleum (Huadong), Qingdao 266580, China; s17090913@s.upc.edu.cn (W.T.); 17862815057@163.com (H.Z.); s19090002@s.upc.edu.cn (X.Y.); s20090094@s.upc.edu.cn (X.Y.); duyg@upc.edu.cn (Y.D.); qd_ldl@upc.edu.cn (D.L.)

**Keywords:** relative humidity, graphene quantum dots, optical fiber sensor, Fabry-Perot

## Abstract

An easily fabricated Fabry-Perot optical fiber humidity sensor with high performance was presented by filling Graphene Quantum Dots (GQDs) into the Fabry-Perot resonator, which consists of two common single mode optical fibers. The relative humidity sensing performance was experimentally investigated by an interference spectrum drift between 11 %RH to 85 %RH. 0.567 nm/%RH sensitivity and 0.99917 linear correlation were found in experiments that showed high sensitivity, good and wide-range linear responding. Meanwhile, its good responding repeatability was demonstrated by two circle tests with increasing and decreasing relative humidity. For investigating the measurement influence caused by a temperature jitter, the temperature responding was experimentally investigated, which showed its linear responding with 0.033 nm/°C sensitivity. The results demonstrate the humidity sensitivity is greatly higher than the temperature sensitivity. The wavelength shift influence is 0.0198 nm with 0.6 °C max temperature jitter in the experiment, which can be ignored in humidity experiments. The fast-dynamic responses at typical humidity were demonstrated in experiments, with 5.5 s responding time and 8.5 s recovering time. The sensors with different cavity lengths were also investigated for their humidity response. All sensors gave good linear responding and high sensitivity. In addition, the relation curve between cavity length and response sensitivity also had good linearity. The combination of GQDs and single mode optical fibers showed easy fabrication and good performance for an optical fiber relative humidity sensor.

## 1. Introduction

Relative humidity (RH) measurements are very important in chemical fields, industrial monitoring, agricultural, pharmaceuticals, environmental monitoring, and so on [1,2,3,4,5,6]. Many interests are attracted by the relative humidity sensor in recent years. Compared with a traditional electrical sensor, the optical fiber sensor has many advantages, such as immunity to electromagnetic interference, high sensitivity, powerful adaptability to a harsh environment, and more [7,8].

Many optical fiber humidity sensors with a different structure or sensitive materials have been developed in recent years. The optical fiber coated with Nafion-crystal was used to measure relative humidity from 40 %RH to 82 %RH in 1997 [9]. A Fabry-Perot (F-P) optical fiber humidity sensor was fabricated by a single mode optical fiber coated by Polyvinyl alcohol (PVA) in 2013 with 0.07 nm/%RH measurement sensitivity from 7 %RH to 91.2 %RH [10]. In the same year, a tapering single mode optical fiber humidity sensor was reported in which the RH was measured from 30 %RH to 90 %RH with 97.76 pm/%RH sensitivity [11]. In 2016, a kind of microfiber coupler coated by adopted silica gel was applied to measure humidity and get 1.6 nm/%RH with the RH values changing from 70% to 86% [12]. Its humidity measurements scale needs to broaden. In 2018, a micro-structured optical fiber with SnO_2_ sputtering deposition was reported with 0.14 rad/%RH sensitivity [13]. In 2019, a relative humidity sensor based on hollow core fiber filled with graphene quantum dots (GQDs) and Polyvinyl Alcohol (PVA) compounds was reported with 117.25 pm/%RH sensitivity from 13.47 %RH to 81.34 %RH [14]. In 2020, Hiba et al. [15] proposed a relative humidity sensor based on a no-core multimode interferometer coated with Al_2_O_3_-PVA composite films and got a maximum sensitivity of 0.587 nm/%RH, but the response linearity in a repeatability experiment was expected to be better. An optical fiber grating RH sensor was also reported, such as polymer-coated fiber Bragg grating [16], which is a long-period fiber grating covered by poly/cobalt chloride [17]. An array of optical fiber long period gratings (LPGs) was demonstrated to monitor relative humidity (RH) changes in the air delivered by a mechanical ventilator operating in different modes with 0.53 nm/RH% sensitivity [18]. The LPG was modified with 10 layers of silica nanoparticles to measure relative humidity. Some special structure optical fiber humidity sensors were reported, such as a U-shaped fiber coated with phenol poly (methyl methacrylate) (PMMA) [19], a microfiber resonator with reduced graphene oxide (RGO) [20], and a D-shaped fiber RH sensor coated with RGO [21]. Many reported sensors have a complex fabrication process, low sensitivity, or an expensive special microstructure optical fiber.

Many kinds of optical fiber humidity sensors are fabricated by various materials coating at the periphery of fiber, such as graphene oxide (GO) [22], MoS_2_ [23], poly (vinyl alcohol) (PVA) [24], poly (methyl methacrylate) (PMMA) [19], silica gel [12], nafion [9], and agarose [25]. The coating material forms a ring structure as a kind of humidity sensitive transducer. Although this kind of humidity response is not direct, the humidity change still influences the intensity, phase, or wavelength of light in the fiber. If the light can be more directly affected by the humidity materials, such as the light transversing in the humidity sensitive materials combined with fiber, then the humidity response may be clear. This requires the humidity sensitive materials to be transparent and adhesive. In our work, graphene quantum dots (GQDs) are used. Therefore, an easily fabricated optical fiber Fabry-Perot humidity sensor composed of a common single mode optical fiber and GQDs is proposed. The optical fiber Fabry-Perot resonator is filled with GQDs. As one part of an F-P resonator, the GQDs will directly affect the light. When the varied refractive index and volume of GQDs are caused by the humidity, the interference spectrum will change. This is used to monitor a humidity change. This sensor has a simple fabrication process and good performance. The humidity response, linearity, repeatability, and the dynamic response are studied in detail, including the temperature response. The experimental results showed its good performance for relative humidity measurements.

## 2. Sensor Fabrication and Sensing Principle

**Figure 1:**
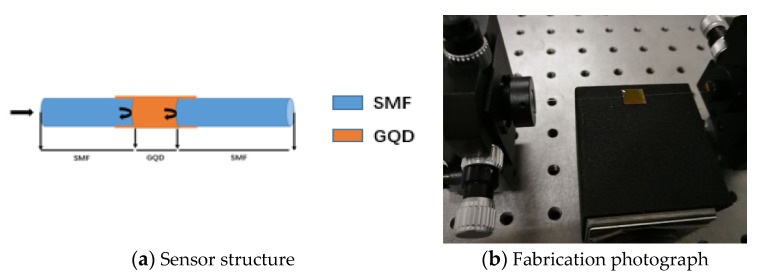
Optical fiber humidity sensor.

The Fabry-Perot cavity structure is fabricated by the end faces of two single-mode fibers as Figure 1a showed. The two end faces are dealt with a fiber cutting knife as the reflection mirrors. The relative position between the two fiber end faces is carefully aligned by the adjustment frame to get a good Fabry-Perot interference spectrum. The interference spectrum is continuously monitored by the MOI (Micro Optics) optical fiber sensor analyzer (SM125) for getting the satisfied spectrum. Then the prepared graphene quantum dots sample is dripped into the optical fiber Fabry-Perot cavity by the dropper. For getting stable adhesion, a little plastic sheet is under the fibers and support the graphene quantum dots liquid drops, as Figure 1b showed. With adhesion and diffusion of the sample droplets, the interference spectrum changed gradually. Therefore, the Fabry-Perot cavity must be fine-tuned again to get the stable and good spectrum before the sample is solidified. Then the F-P cavity with GQDs is steadily placed for more than 24 h. After the sample is dried out and solidified, an optical fiber humidity sensor is obtained. In the process of the making sensor, the stereomicroscope is used to observe the cavity and measure the cavity length.

As a new carbon nanomaterial, graphene quantum dots have the characteristics of graphene and quantum dot materials. The oxygen-containing functional groups on GODs’ surface give it good hydrophilic properties. The surface also has an ultra-high active carrier, which easily absorbs the external water molecules. Compared with traditional quantum dots materials, graphene quantum dots have good water solubility, high biocompatibility, low biological toxicity, and other advantages. Graphene quantum dots can be synthesized by thermal cracking of citric acid. The principle of the preparation method is to adjust the degree of carbonization of citric acid. The carboxyl functional group carried by the citric acid itself undergoes self-dehydration condensation at a high temperature to form graphene quantum dots. The advantages of the pyrolysis method include cheap raw materials, low equipment requirements, simple steps, and high experimental safety. Therefore, we use this method to get graphene quantum dots.

We place some citric acid in a beaker. Then it is heated from room temperature to 180 °C. Until the color becomes orange-red, the product is made of graphene quantum dots. The beaker is taken out from the furnace and cooled some minutes. We put 2.5 g of graphene quantum dots powders and 50 mL of deionized water together into a beaker, and stirred slightly with a glass bar. Then a magnetic stirrer is used to get a good stirring effect for 5 min. Finally, we obtained a graphene quantum dots solution with the concentration of 50 mg/mL.

The optical fiber Fabry-Perot cavity is filled with QODs, as Figure 1a showed. The light in the fiber is reflected at two interfaces between the fiber ends and QODs. Then, the interference spectrum is got by these two reflecting beams. The optical path difference between two beams is shown as:

(1) $$\Delta {L\;=\;2n}_{e}d$$

where $n_{e}$ is the effective refractive index of QODs, and d is the cavity length. The interference intensity is shown as:

(2) $${I\;=\;I}_{0}{[R}_{1}{\;+\;R}_{2}\;+\;2\sqrt{R_{1}R_{2}}\cos(\frac{2\pi \Delta L}{\lambda }\;+\;\emptyset_{0})]$$

where I_0_ is incidental light intensity of the optical fiber sensor, and R_1_, R_2_ is the reflectivity of two reflectors, respectively. *λ* is the light wavelength, and ϕ_0_ is the phase constant. If the relative humidity changed, the refractive index of GQDs will change because GQDs adsorb water molecules [26]. The GQDs layers will swell after the interaction with water molecules [26]. Then the optical path difference Δ*L* will also change and lead to the Fabry-Perot interference spectrum shift. The relationship between the spectrum shift and relative humidity can be measured by a spectrometer.

The optical path difference change is shown as:

(3) $$\Delta L=\;2(\Delta n_{e}d\;+\;n_{e}\Delta d)=L(\frac{\Delta n_{e}}{n_{e}}\;+\;\frac{\Delta d}{d})$$

where *n_e_* is the effective refractive index of medium in F-P cavity and *d* is the F-P cavity length.

When the optical path difference satisfied interference strengthening conditions, the light intensity reaches the maximum value, and the peak wavelength of reflected spectrum is shown in the equation below.

(4) $$\lambda_{m}=\frac{2n_{e}d}{m}$$

Therefore, $\lambda_{m}$ is determined by d and *n_e_*, *m* is an integer. In addition, the interference spectrum shift is shown below.

(5) $$\frac{\Delta \lambda_{m}}{\lambda_{m}}=\frac{\Delta n_{e}}{n_{e}}+\frac{\Delta d}{d}$$

When the RH change, the reflective spectrum shifts. The RH can be measured by monitoring the peak wavelength shift.

## 3. Experiments

### 3.1. Experimental Setup

**Figure 2:**
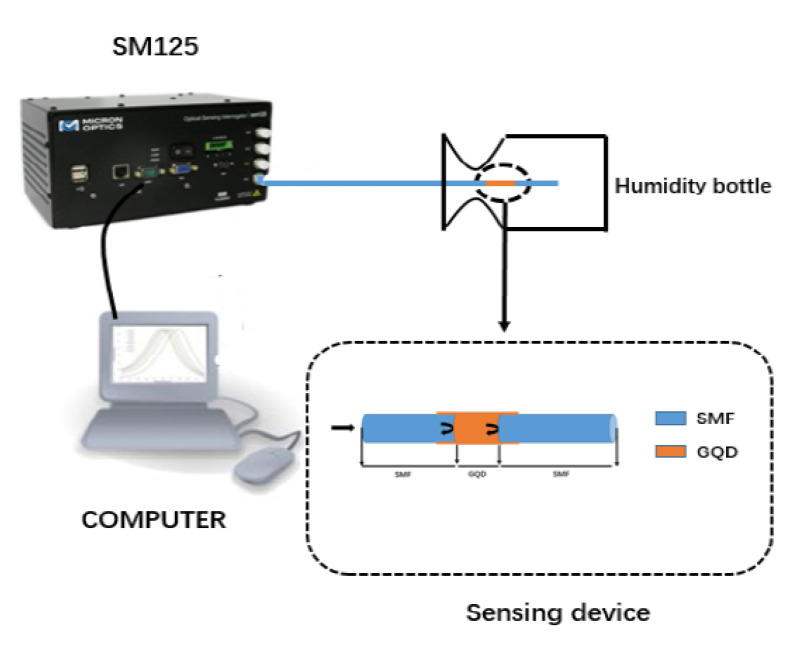
Experimental setup.

The experimental setup is shown in Figure 2. A Micron Optics SM 125 optical sensing interrogator is connected to an optical fiber sensor. Its wavelength range is 1510–1590 nm, the scanning frequency is 2 Hz, the wavelength accuracy and stability reach 1 pm, and the dynamic measurement range is 50 dB. The saturated salt solution bottles can give a different humidity environment from 11 %RH to 85 %RH. The hygrometer is also used to monitor and calibrate the humidity values. The sensor was naturally placed in the experiment. We moved the humidity bottle to bring the sensor into a different humidity environment. When the sensor is placed in the bottle, the GQDs will absorb water molecules because of its hygroscopicity. Then its refractive index and size change with different ambient humidity. Therefore, the spectrum shift is observed.

### 3.2. Humidity Responding Experiments and Discussions

**Figure 3:**
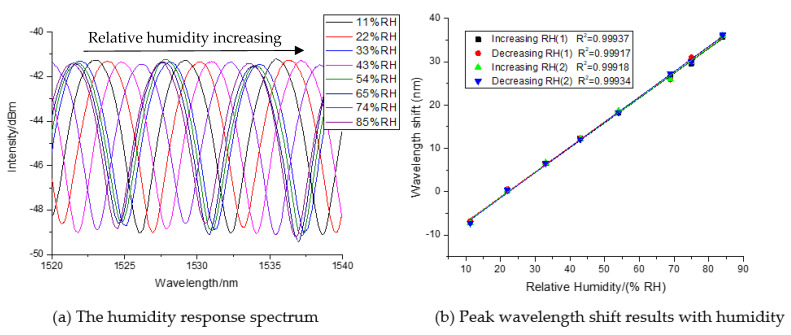
Humidity response results with a different humidity.

The humidity measurement experiments were executed at 26.8 °C. The relative humidity changed from 11% to 85%. The sensor is fabricated by 50 mg/mL QODs solution and two single mode optical fibers with a 405-μm cavity length of the Fabry-Perot resonator. When the optical fiber sensor is placed in different humidity environments, the Fabry-Perot interference spectrum will shift, as Figure 3a showed. The response spectrums with a different humidity are measured, respectively, at 11 %RH, 22 %RH, 33 %RH, 43 %RH, 54 %RH, 65 %RH, 74 %RH, and 85 %RH. With the increase of relative humidity, the interference spectrum pans to the right, which is contrary to the left with a decreasing humidity value. Clearly, the spectrum shift degree is connected to the RH value.

According to the spectrum shift data, we can get the relative humidity response curves, as Figure 3b showed. A wavelength shift value is negative because the value is lower than the ambient initial relative humidity value. In Figure 3b, it can be seen that the interferometric wavelength shift value has a good linear relationship with the relative humidity from 11%RH to 85%RH. The linearity of the measurement results in the process of humidity rising to 0.99937, and that, in the process of humidity, falling to 0.99917 during the first test circle. The humidity responding sensitivity reaches 0.547 nm/%RH in the process of relative humidity rising, and 0.567 nm/%RH in the process of relative humidity decreasing. The experimental results showed that the sensor has high humidity response sensitivity. For investigating its humidity responding repeatability, the humidity responding curves are measured again with an up and down humidity value, as Figure 3b showed. Clearly, the wavelength drift curves of two circles showed that all response curves are well coincided. Therefore, the sensor also has good repeatability in a wide humidity range.

In the experiments, we found a little temperature jitter. As we know, the optical fiber Fabry-Perot sensor usually responds with the temperature. The spectrum fluctuation caused by the temperature may be one source of experimental errors. For investigating this influence, the temperature responding behavior is also investigated.

**Figure 4:**
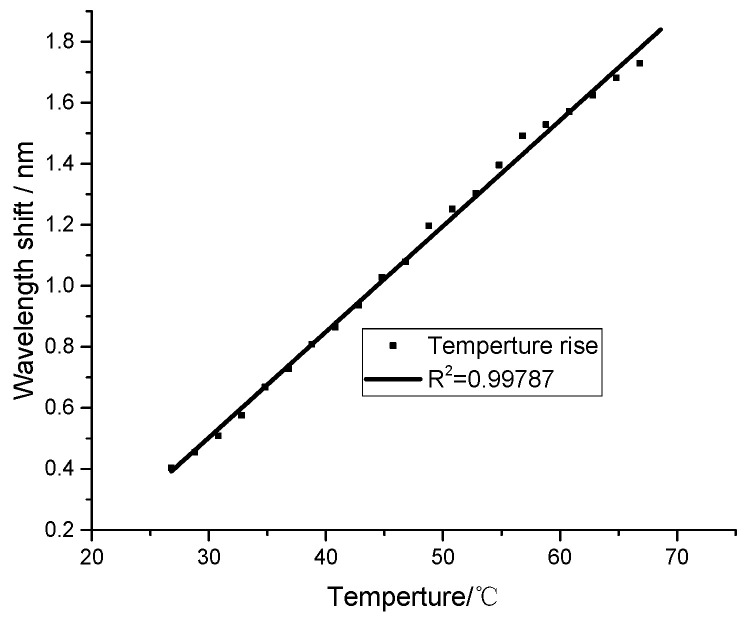
Temperature responding results.

Using the former experimental system, we placed the humidity sensor in different temperature surroundings with a controlled heating system. The initial ambient temperature is 26.8 °C with 22% relative humidity. The interference spectrum shift is investigated from 26.8 °C to 68.6 °C. Therefore, the experimental results of the peak wavelength shift values varied with the temperature, as shown in Figure 4. We can see the wavelength shift of the interference spectrum has a good linear relationship with the temperature, with 0.99787 linear correlation. The temperature responding sensitivity is 0.033 nm/°C. Compared with the humidity sensitivity of 0.547 nm/%RH and 0.567 nm/%RH, the sensor has lower temperature response sensitivity. This is very helpful in dealing with the cross-response of humidity and temperature. Some points are off-line because of the slightly fluctuated relative humidity in the experiments.

**Figure 5:**
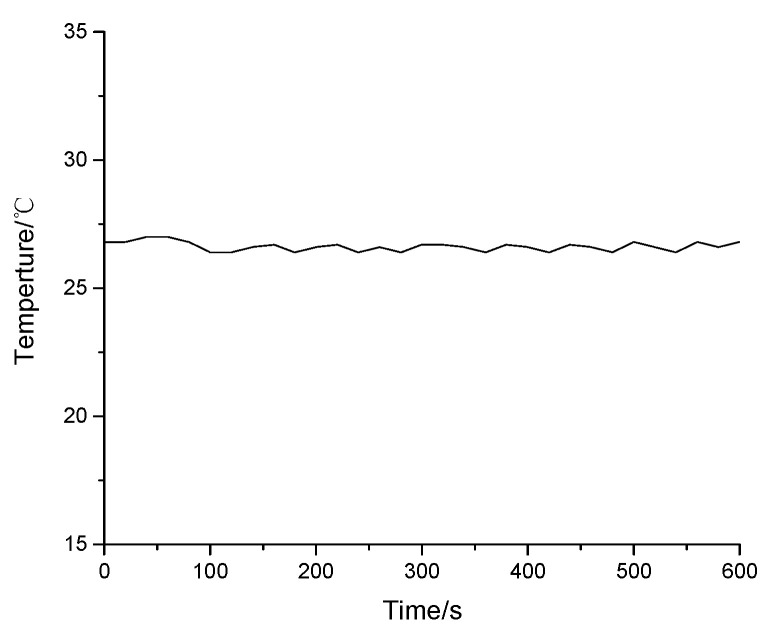
Temperature monitoring data.

Compared with the humidity responding results in Figure 3, the temperature influence can be evaluated. The temperature change of the humidity responding test is shown in Figure 5. The temperature is nearly kept at 26.8 °C. The max temperature difference in measurements is 0.6 °C. This means the max wavelength shift caused by a temperature change is 0.0198 nm. The influence to humidity responding results is very slight on a whole measurement scale, which can be ignored.

### 3.3. Dynamic Responding of the Sensor

**Figure 6:**
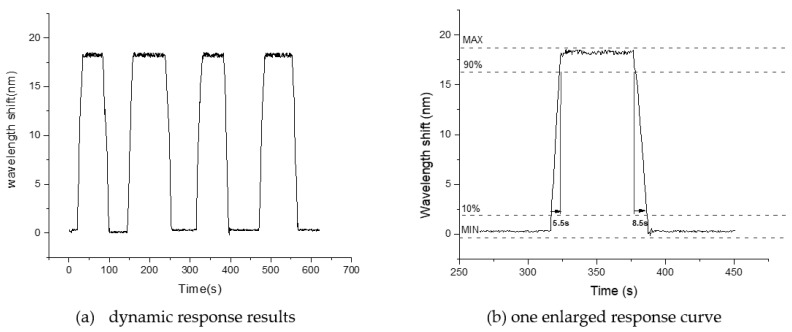
The dynamic responding experimental curves of 54%RH.

One important application of the relative humidity sensor is dynamic humidity monitoring. Therefore, the dynamic responding of the sensor is also investigated. In experiments, the humidity bottle is immediately moved to let the sensor in or out of the bottle. The spectrum is auto saved by the MOI optical fiber sensor analyzer (SM125) every 0.5 s. By analyzing the spectrum shift, we got the dynamic responding data of this humidity sensor. The peak wavelength shift results at a typical humidity value of 54 %RH, which is shown in Figure 6.

As Figure 6a showed, the dynamic responding measurement is repeated four times in 625 s. Therefore, four responding circle curves give us the information of responding time and recovery time, as the rising edge and falling edge showed in Figure 6a. We also see the sensor has good repeatability of dynamic responding. Figure 6b is the enlarged image of one dynamic responding curve. As the figure showed, the sensor has fast responding performance, with 5.5 s responding time and 8.5 s recovering time. The recovering time is longer than the responding time because of the QODs humidity sensitive characteristic. The recovering time is closely related to the removing speed of the water molecule. For QODs, the water molecule absorption speed is different with the water molecule detachment speed.

### 3.4. The Cavity Length Influence

**Figure 7:**
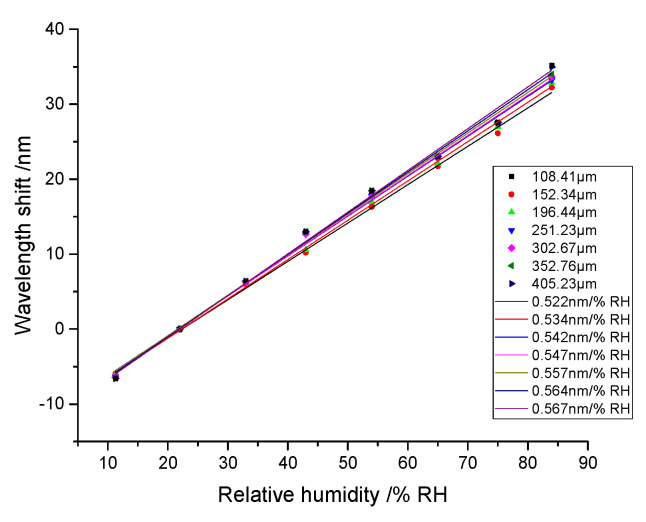
The relative humidity response curves of different Fabry-Perot cavity lengths.

The F-P cavity parameters directly affect the interference spectrum, especially the cavity length. To investigate the influence on the humidity response, the humidity sensing experimental results are also obtained. Seven RH optical fiber sensors with a different cavity length are all evaluated. In experiments, the cavity length is 108.41 μm, 152.34 μm, 196.44 μm, 251.23 μm, 302.67 μm, 352.76 μm, and 405.23 μm, respectively. All the humidity responding curves are shown in Figure 7.

**Figure 8:**
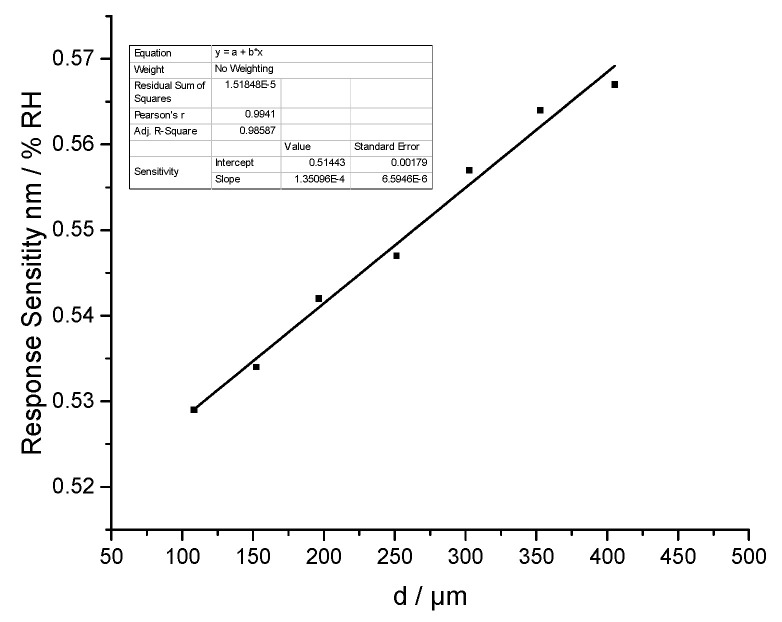
The humidity response sensitivity with a different Fabry-Perot cavity length.

As Figure 7 showed, each sensor had good linear humidity responses from 11 %RH to 85 %RH. Therefore, its linear responding characteristic is credible even if cavity length changes. The humidity response sensitivity of each sensor is 0.522 nm/%RH, 0.534 nm/%RH, 0.542 nm/%RH, 0.547 nm/%RH, 0.557 nm/%RH, 0.564 nm/%RH, and 0.567 nm/%RH, respectively. The responding sensitivity increased with a bigger cavity length. Figure 8 showed the relationship between the sensitivity and cavity length. This curve also has good linearity with 0.9941 linear Pearson’s r value. Therefore, the sensitivity proportionally increased with cavity length. Large cavity length is helpful to improve the humidity sensitivity.

## 4. Discussion

Table 1 showed the performance comparison of some optical fiber humidity sensors. The sensor of the reference [27] is more sensitive to humidity but only at a low humidity value. The responding sensitivity of Reference [28] is clearly different between low and high humidity. Therefore, its linear responding ability needs to be improved. In addition, the side-polished twin-core fiber induced a complicated fabrication and high cost. Our sensor linear responding range is wider than the sensors from References [22,27,28]. Meanwhile, our sensor is more sensitive to humidity than the sensors from References [8,14,22].

Table 2 is the comparison of other optical fiber RH sensors based on different graphite materials. We can see that our senor is more sensitive to humidity. Its linear responding scale and correlation coefficient are also better than other sensors in Table 2.

According to the previous comparison, this configuration has high sensitivity with good linearity, wide linear responding scale, easy fabrication, and a fast-dynamic response.

## 5. Conclusions

An optical fiber Fabry-Perot relative humidity sensor composed of QODs and single-mode fibers has been fabricated and experimentally investigated. The interference peak wavelength shift can be tracked by MOI SM125 in the experiments. The humidity responding experiments have been operated in a wide humidity range (11 %RH–85 %RH), and given 0.567 nm/%RH sensitivity and 0.99917 linear correlation. The humidity measurements were repeated at two humidity changing circles, which showed its good responding repeatability. For investigating the influence caused by a temperature jitter, the temperature responding characteristic was also experimentally investigated. The temperature responding curve had 0.033 nm/°C sensitivity and 0.99787 linear correlation. The influence to wavelength shift is 0.0198 nm with a 0.6 °C max temperature jitter in the entire experimental process. The temperature responding sensitivity is greatly lower than the humidity. Furthermore, the sensor has a stable and fast dynamic humidity response, which was discovered through the dynamic measurement experiments. We obtained a 5.5 s responding time and an 8.5 s recovering time at 54 %RH. In short, this kind of sensor has high sensitivity, an easy fabrication process, low cost, a wide linear measuring scale, good repeatability, and a rapid dynamic response. Some applications need a high performance and low-cost relative humidity sensor. This sensor indicated the possibility of easy implementation.

## Figures and Tables

**Table 1:** Comparison of other fiber optic humidity sensors.

Method	Humidity Sensitive Material	RH/%	Sensitivity	Response and Recovery Time/s	Reference
Microstructured polymer fiber bragg grating	Polycarbonate	20~90	0.00731 nm/%RH	10.5, 25	[8]
Long-period grating	Titanium dioxide	0~10 10~20	1.027 nm/%RH 1.453 nm/%RH	19, 27.1	[27]
Side-polished twin-core fiber	Graphene-oxide	40~75 60~62.1	2.72 nm/%RH 3.76 dB/%RH	3.6, 6.4	[28]
wheel side-polishing and evaporation	graphene-oxide	32~85 85~97.6	0.145 nm/%RH 0.915 nm/%RH	2.73, 7.27	[22]
F-P interferometer	PVA-GQDs	11.3~83.4	0.11725 nm/%RH	4.3, 11.5	[14]
F-P interferometer	GQDs	11~84	0.567 nm/%RH	5.5, 8.5	Our work

**Table 2:** Comparison of other optical fiber RH sensors based on different graphite materials.

Material	Measurement Range (%RH)	Sensitivity	Correlation Coefficient	Reference
Graphene oxide	10–80	0.129 dB/%RH	99%	[29]
Graphene oxide	32–85	0.145 nm/%RH	99.6%	[22]
	85–97.6	0.915 nm/%RH	98.7%	
Reduced graphene oxide	30–50	0.0537 nm/%RH	96.33%	[20]
Reduced graphene oxide	75–95	0.31 dB/%RH	98.2%	[21]
Graphene oxide/PVA	25–80	0.193 dB/%RH	99.1%	[30]
Graphene oxide/PVA	40–60	0.0128 dB m/%RH	96.786%	[31]
PVA/GQDs	11.3–81.34	0.11725 nm/%RH	99.83%	[14]
GODs	11-85	0.567 nm/%RH	99.92%	Our work

## Data Availability

Not applicable.
